# Relationship between intrathrombotic appearance of HSP27 and HSP70 and thrombus ages in a  murine model of deep vein thrombosis

**DOI:** 10.1038/s41598-023-48987-5

**Published:** 2023-12-16

**Authors:** Mizuho Nosaka, Yuko Ishida, Yumi Kuninaka, Akiko Ishigami, Akira Taruya, Emi Shimada, Yumiko Hashizume, Hiroki Yamamoto, Akihiko Kimura, Fukumi Furukawa, Toshikazu Kondo

**Affiliations:** 1 Department of Forensic Medicine, Wakayama Medical University, 811-1 Kimiidera, Wakayama, 641-8509 Japan; 2 Department of Cardiovascular Medicine, Wakayama Medical University, 811-1 Kimiidera, Wakayama, 641-8509 Japan; 3 Takatsuki Red Cross Hospital, 1-1-1 Abuno, Takatsuki, Osaka 569-1096 Japan

**Keywords:** Immunology, Coagulation system

## Abstract

Heat shock proteins (HSPs) are molecular chaperones whose primary function is cytoprotection, supporting cell survival under (sub) lethal conditions. They have been implicated in various diseases such as inflammatory diseases and cancer due to their cytoprotective and immunomodulatory effects, and their biological mechanisms have been studied. Central family members include, HSP27, which is induced by various stimuli such as heat shock, hypoxia, hyperoxia, ultraviolet exposure, and nutritional deficiency, and HSP70, which is homeostatically expressed in many organs such as the gastrointestinal tract and has anti-cell death and anti-inflammatory effects. In this study, HSP27 and HSP70 were investigated during thrombus formation and dissolution in a deep vein thrombosis model by immunohistochemistry to determine their involvement in this process and whether their expression could be used as a forensic marker. In the process of thrombus formation and lysis, HSP27 and HSP70 were found to be expressed by immunohistochemical analysis. The role of inhibitors of HSP27 and HSP70 in the pathogenesis of thrombosis in mice was also investigated. When HSP27 or HSP70 inhibitors were administered, thrombi were significantly smaller than in the control group on day 5 after inferior vena cava ligation, indicating pro-thrombotic effects HSP27 and HSP70. If HSP27- or HSP70-positive cells were clearly visible and easily identifiable in the thrombus sections, the thrombus was presumed to be more than 10 days old. Thus, the detection of intrathrombotic HSP27 and HSP70 could forensically provide useful information for the estimation of thrombus ages. Collectively, our study implied that both HSP27 and HSP70 might be molecular targets for thrombus therapy and that the detection of HSP-related molecules such as HSP27 and HSP70 could be useful for the determination of thrombus ages.

## Introduction

Heat shock proteins (HSPs) are a family of proteins that are produced in cells by various stimuli, such as high temperature, hypoxia, hyperoxia, UV exposure, nutrient deprivation, and other stressors, and their main role is to maintain cellular homeostasis and to promote cell survival. HSP27 is known as the HSPB family that are ubiquitously present in mammalian cells and are encoded by the heat shock protein beta-1 (HSPB1) gene^1^. HSP27 exhibits chaperone-like activity, prevents aggregation of partially unfolded proteins, protects cells from oxidative stress by scavenging reactive oxygen species (ROS), and protects cells from apoptosis in response to stress conditions by blocking cytochrome c and inhibiting caspase-3 activation^2,3^. HSP70 is a member of the HSPA family, and it has been assumed to protect the cell via its chaperone functions. Indeed, overexpression of HSP70 appears to prevent protein aggregation and redistribution of ubiquitin^4^.

In this study, we aimed to establish a new index for determining the degree of thrombus obsolescence by immunohistochemically analyzing the dynamics of heat shock proteins over time in order to find the possibility that stress proteins are involved in the process of thrombus formation and dissolution^5–9^.

From a forensic perspective, pulmonary thromboembolism resulting from deep vein thrombosis (DVT) is one of the leading causes of sudden unexpected death. We have elucidated the pathogenesis of DVT from both forensic and experimental pathology^10,11^. Later, using knockout mice, we found that cytokines such as IFN-γ, TNF-α and IL-6 play an important role in the resolution of DVT^12–14^. Determining the elapsed time taking for a wound to persist or for a thrombus to form is one of the important matters^15–21^. Therefore, we reported several specific cells and markers for thrombus age estimation^10,11^.

The HSP expression under heat stress has already been demonstrated in myocardium, lung and kidney tissues and HSPs can be visualized with immunohistochemical staining^22–24^. Not only fire deaths but also cardiac deaths, mechanical asphyxiation and acute lung injury, HSPs were detected^24^. In the present study, we investigated the expression of HSPs under hypoxic conditions of blood flow stasis. We immunohistochemically examined intrathrombotic appearance of HSP27 and HSP70 and discussed the possibility of their application to thrombus age estimation in forensic practices.

## Results

### Intrathrombotic HSP27 and HSP70 expressions after the inferior vena cava (IVC) ligation

**Figure 1:**
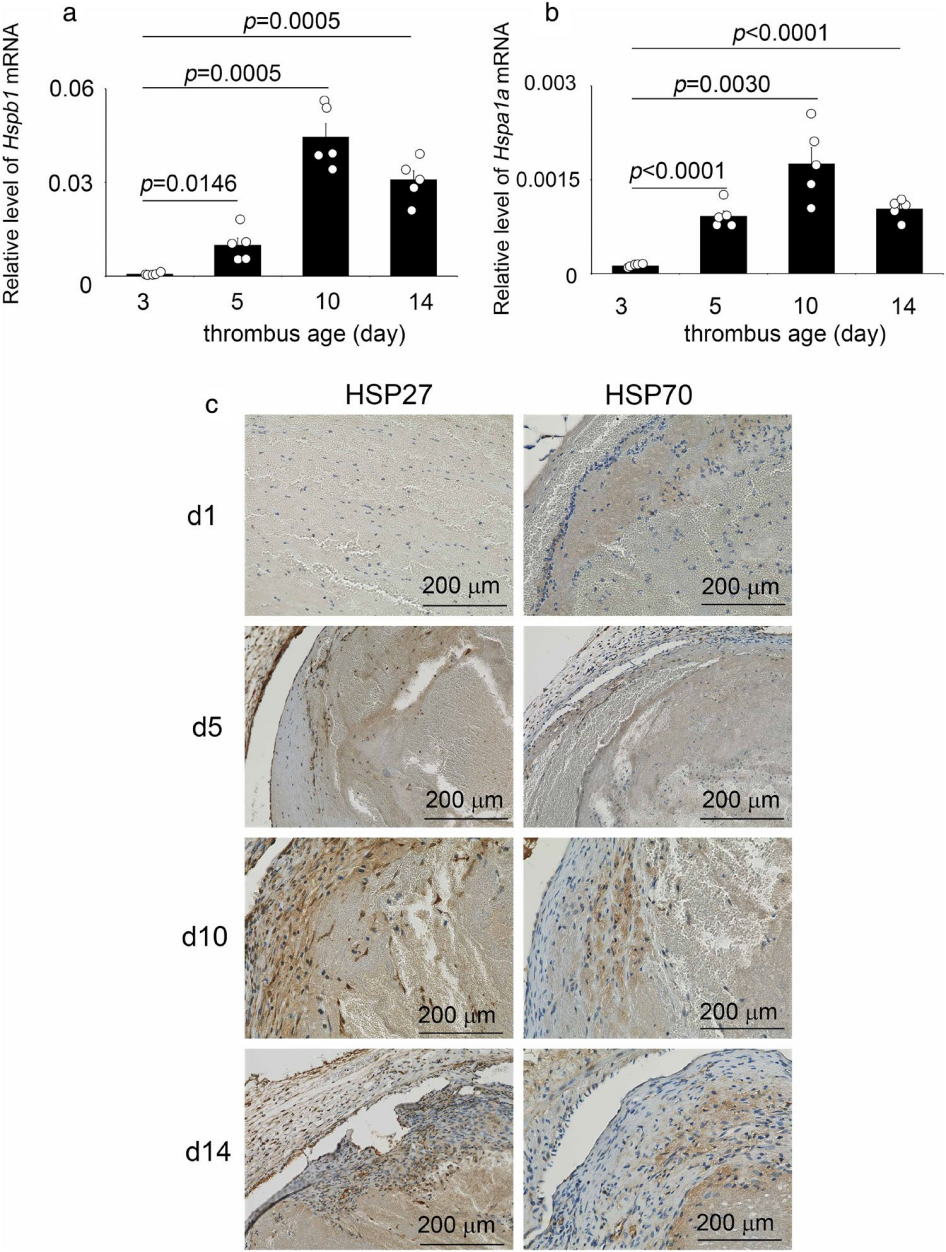
Intrathrombotic expressions of HSP27 and HSP70 in mice. (**a** and **b**) Intrathrombotic gene expressions of *Hspa1a* and *Hspb1* were examined by real-time RT-PCR in mice after inferior vena cava (IVC) ligation. *Hspb1*: murine HSP27 gene; *Hspa1a*: murine HSP70 gene. n = 5 in each group. (**c**) Immunohistochemical analysis of HSP27 and HSP70 in mouse thrombi. Representative results were shown (n = 5).

The intrathrombotic gene expressions of *Hspb1* (*Hsp27)* and *Hspa1a* (*Hsp70*) were observed on day 3, then increased and was highest on day 10, and then decreased (Fig. 1a and b). The trend of increase or decrease in the expression of both genes was consistent with that of the number of positive cells determined from immunohistochemistry (Fig. 1c).

### Intrathrombotic distributions of HSPs and macrophages

**Figure 2:**
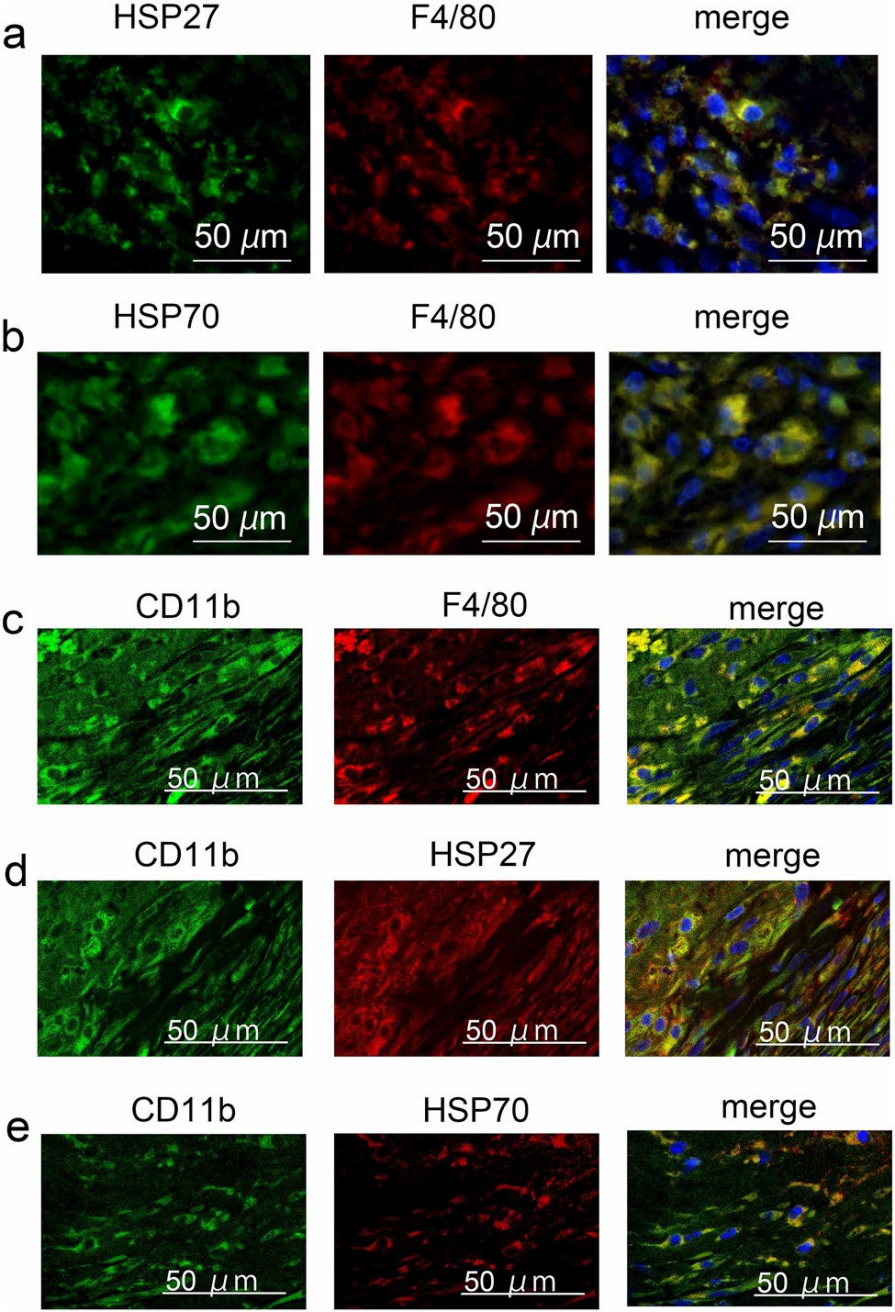
Double-color immunofluorescence analysis of HSPs and F4/80 or CD11b in the 14-days-thrombi. (**a**) HSP27 (green), F4/80 (red) and DAPI (blue); (**b**) HSP70 (green), F4/80 (red) and DAPI (blue); (**c**) CD11b (green), F4/80 (red) and DAPI (blue); (**d**) CD11b (green), HSP27 (red) and DAPI (blue); (**e**) CD11b (green), HSP70 (red) and DAPI (blue).

**Figure 3:**
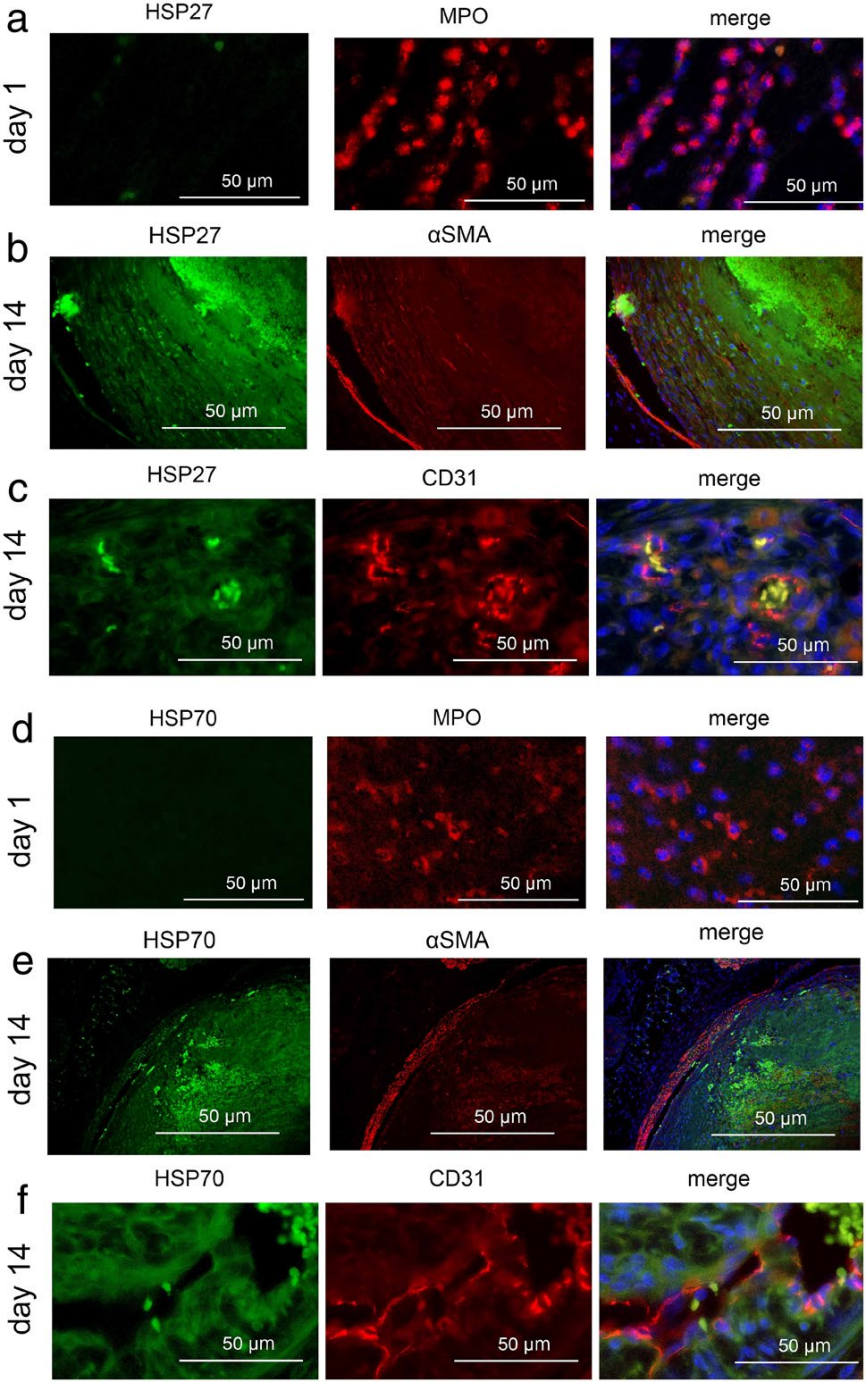
Double-color immunofluorescence analysis of HSPs and MPO, α-SMA, or CD31 in the thrombi at the indicated time points. (**a**) HSP27 (green), MPO (red) and DAPI (blue); (**b**) HSP27 (green), α-SMA (red) and DAPI (blue); (**c**) HSP27 (green), CD31 (red) and DAPI (blue); (**d**) HSP70 (green), MPO (red) and DAPI (blue); (**e**) HSP70 (green), α-SMA (red) and DAPI (blue); (**f**) HSP70 (green), CD31 (red) and DAPI (blue).

Fluorescence double staining analysis showed that the intrathrombotic localizations of HSP27^+^ or HSP70^+^ cells and F4/80^+^ macrophages were consistent (Fig. 2a and b). In addition, most F4/80^+^ cells expressed CD11b (another macrophage maker), and CD11b^+^ cells expressed both HSP27 and HSP70 (Fig. 2c–e). In contrast, MPO^+^ neutrophils, α-SMA^+^ fibroblasts, and CD31^+^ endothelial cells did not express HSPs (Fig. 3). These results suggested that macrophages were the main producers of both HSP27 and HSP70 in the thrombi.

### Intrathrombotic appearance of HSP27^+^ or HSP70^+^ cells

**Figure 4:**
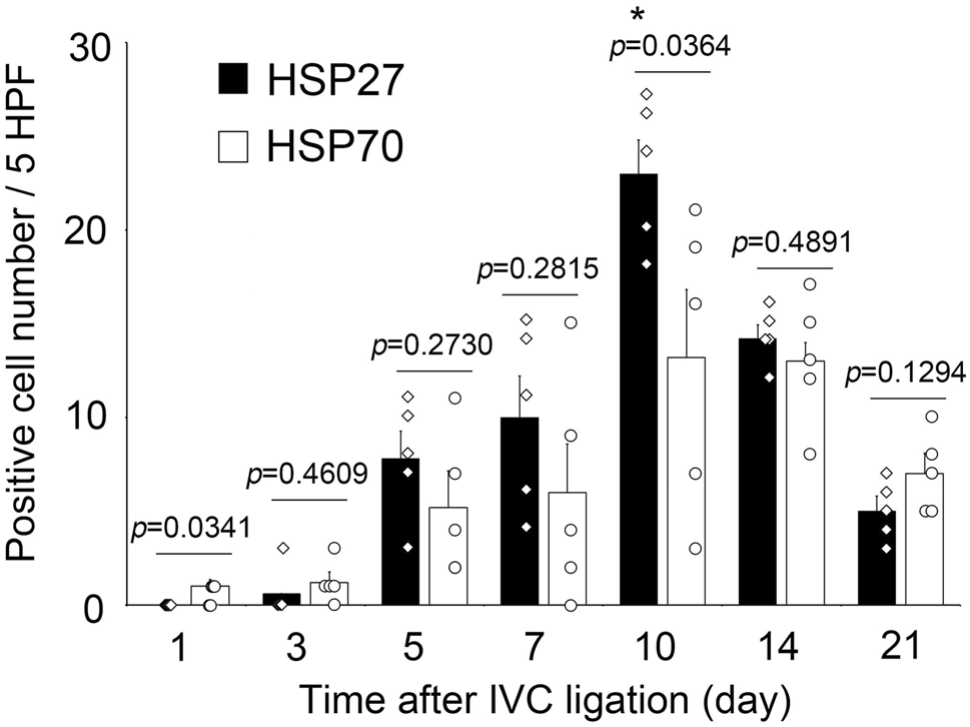
Changes in intrathrombotic HSP27^+^ cell and HSP70^+^ cell numbers after IVC ligation. Both positive cell number of HSP27 and HSP70 showed maximum values on day 10, but HSP27 was significantly abundant than HSP70. n = 5 in each group.

In the sub-acute and chronic phase thrombi, the HSP27^+^ and HSP70^+^ cells were detected (Fig. 1c and Table 1). Until day 10, when the inflammatory response is dominant in thrombus formation^25^, the number of HSP27^+^ cells exceeded the number of HSP70^+^ cells (Fig. 4). The HSP27^+^ cells were more abundant than HSP70^+^ cells until the thrombus entered the chronic phase, and by day 10, HSP27^+^ cells were significantly more numerous than HSP70^+^ cells, but by day 14, both HSP27 and HSP70 were almost the same. These results suggest that the kinetics of HSP27 and HSP70 expression in thrombus can be a useful index for determining the degree of obsolescence of thrombus even in autopsy cases.

**Table 1:** Mean intrathrombotic HSP27^+^ cells and HSP70^+^ cell numbers (n = 5).

Thrombus age (day)	HSP27^+^ cells Mean ± SEM (range)	HSP70^+^ cells Mean ± SEM (range)
1	0	1.00 ± 0.37 (0–2)
3	0.60 ± 0 .00 (0–3)	1.20 ± 0.56 (0–3)
5	7.80 ± 1.48 (0–11)	5.20 ± 1.95 (2–11)
7	10.00 ± 2.22 (4–15)	6.00 ± 2.59 (0–15)
10	23.00 ± 1.80 (18–27)	13.20 ± 3.62 (3–21)
14	14.20 ± 0.73 (12–16)	13.00 ± 0.99 (8–17)
21	5.00 ± 0.82 (3–7)	7.00 ± 1.10 (5–10)

### Both HSP27 and HSP70 inhibitors reduced thrombi size in mice

**Figure 5:**
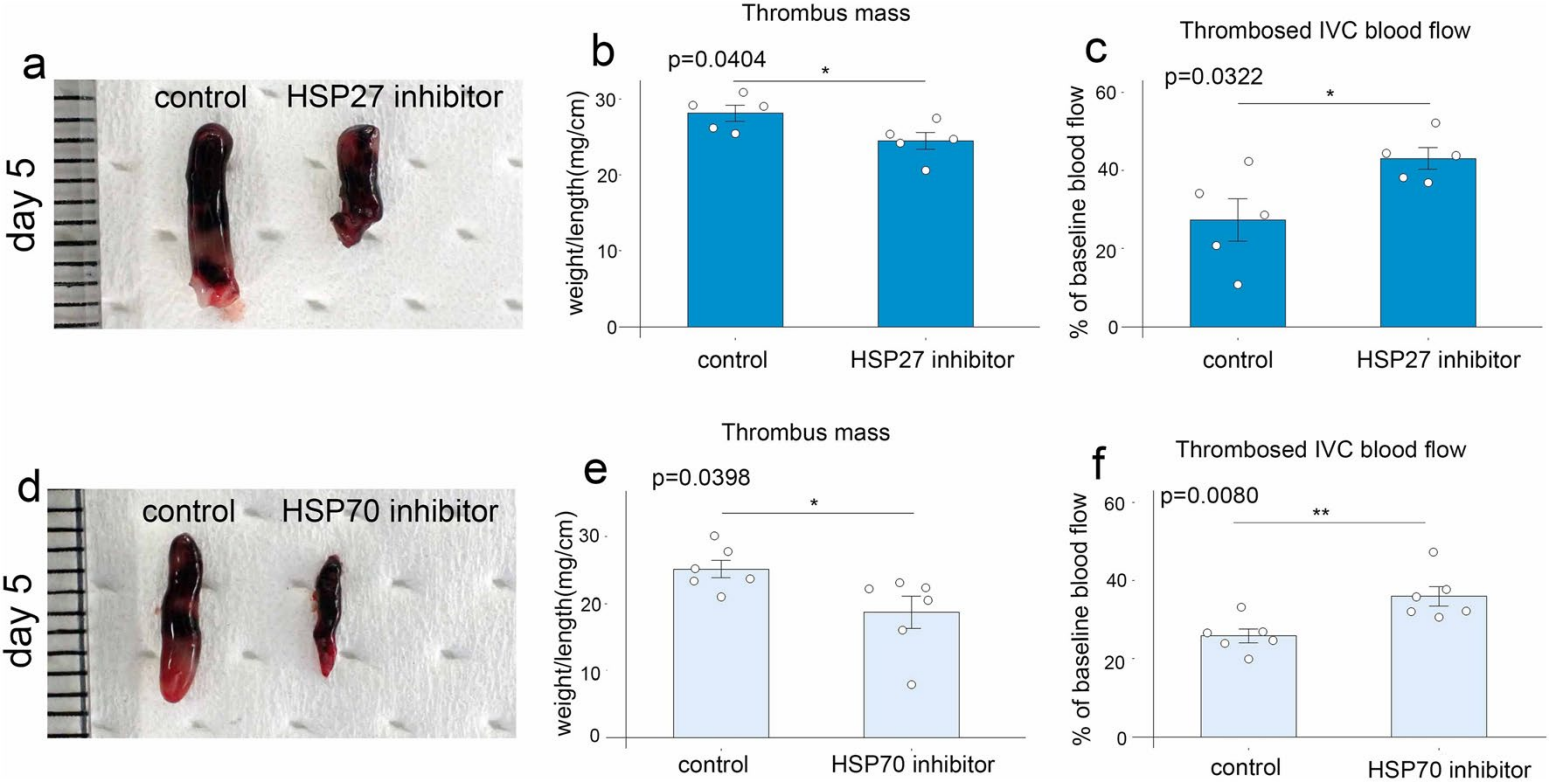
The effects of HSP27 and HSP70 inhibitor in murine thrombosis. (**a**) Macroscopic appearance of venous thrombi obtained from mice treated with HSP27 inhibitor or PBS as controls. Representative results from five independent animals are shown. Thrombus mass (**b**) and thrombosed blood flow (**c**) were measured at 5 days after IVC ligation. (**d**) Macroscopic appearance of venous thrombi obtained from mice treated with HSP70 inhibitor or PBS as controls. Representative results from six independent animals are shown. Thrombus mass (**e**) and thrombosed blood flow (**f**) were measured at 5 days after IVC ligation. All values represent the mean ± SEM (n = 5–6 animals). **p < 0.01, *p < 0.05 vs. control.

In another experiment, mice were treated with HSP27 or HSP70 inhibitors to explore the role of HSPs. Five days after IVC ligation, mice treated with pharmacological inhibitors of HSP27 or HSP70 developed smaller thrombi compared to PBS-treated control mice (Fig. 5a,b,d,e). Furthermore, blood flow was increased in mice treated with inhibitors compared to control mice (Fig. 5c and f). These observations suggest that HSP27 and HSP70 may play an important role in the pathogenesis of thrombosis.

### Estimation of thrombus age using human samples

**Figure 6:**
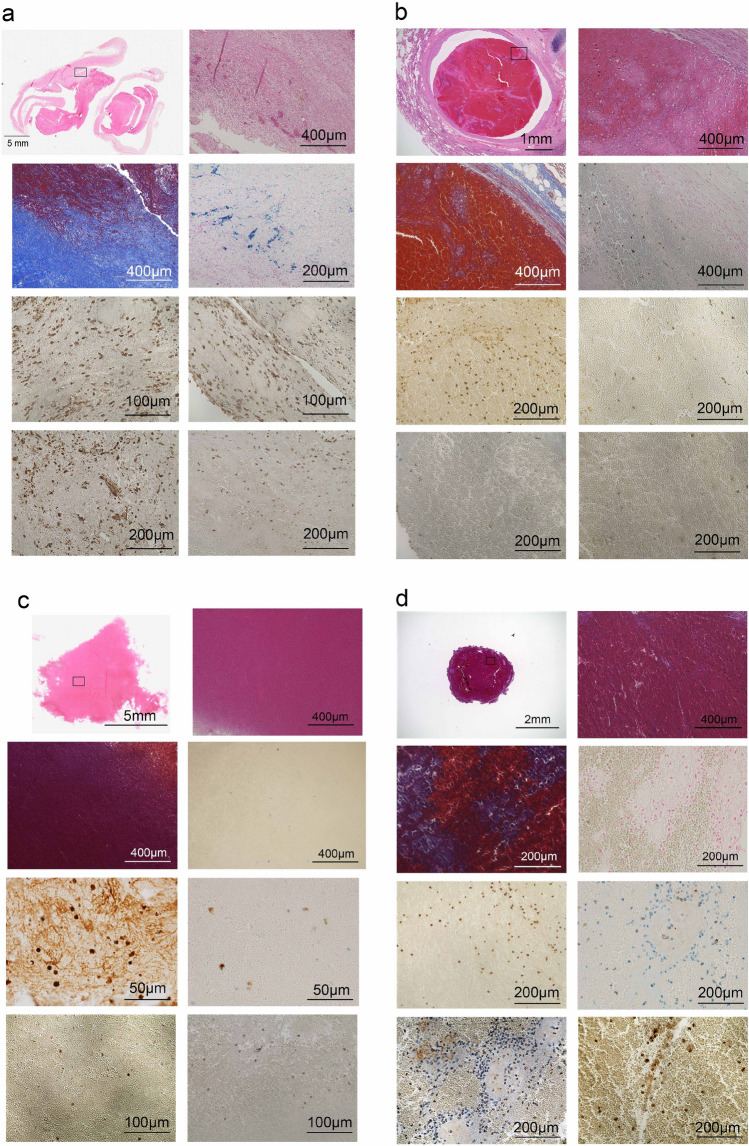

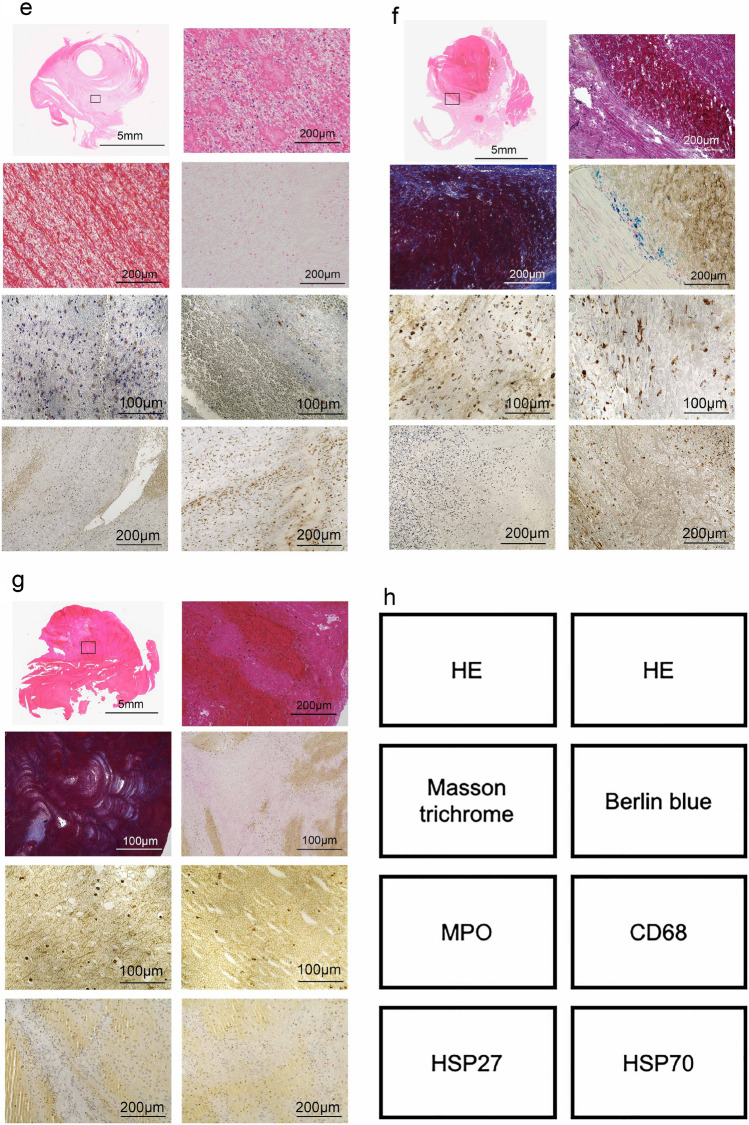
Histopathological and immunohistochemical images of human thrombi. As show in Table 2, case 1 (**a**); case 2 (**b**); case 3 (**c**); case 4 (**d**); case 5 (**e**); case 6 (**f**); and case 7 (**g**). (**h**) Staining indication panels in (**a**) to (**c**).

In the next step, thrombus samples collected from human were analyzed histopathologically and immunohistochemically to estimate thrombus age (No. 1 to 7 in Fig. 6 and Table 2). As an example, No.1, the thrombus showed that the collagen content area was approximately 45% and number of hemosiderin-positive cells was 33 (Fig. 6a and Table 2). In addition, the ratio of MPO-positive neutrophils to CD68-positive macrophages (N/M ratio) referred to 0.9 (Fig. 6a and Table 2). Moreover, the number of HSP70-positive cells was markedly lower than that of HSP27-positive cells. (Fig. 6a and Table 2) Taken together, these observations suggest that the human thrombus No. 1 may be 7–10 days old based on our findings^25,26^. The thrombi from No. 2 to 7 were analyzed in the same way and the thrombus age was estimated (Fig. 6b–g and Table 2).

**Table 2:** Determination of human thrombus age.

Case	Collagen content area (%) collagen area/thrombus area (pixels)	Hemosiderin-positive cell number	N/M ratio	HSP27^+^ cells	HSP70^+^ cells	Estimated thrombus age (day)
No. 1	85.1/186.7 = 45.6	33	52/56 = 0.9	27	17	7–10
No. 2	0.3/15.7 = 1.9	0	63/13 = 4.8	5	9	1–3
No. 3	0.6/102.5 = 0.6	3	81/15 = 5.4	3	2	1–3
No. 4	0.2/5.4 = 3.7	0	97/35 = 2.8	10	12	3–5
No. 5	0.7/86.7 = 0.8	0	47/6 = 7.8	7	13	1–3
No. 6	4.2/41.8 = 10.0	12	44/17 = 2.6	6	11	3–5
No. 7	4.4/195.4 = 2.2	0	31/8 = 3.9	6	3	1–3

## Discussion

The expression of HSPs in the lungs, myocardium, and kidneys under heat stress was clear, and their expression was a useful indicator for estimating the cause of death and time since death^22–24^. To elucidate the types of stresses under which HSPs are expressed, we confirmed their expression in experimental thrombi. HSPs were identified not only in experimental thrombi but also in thrombi found postmortem, suggesting that the relationship between thrombi and HSPs is also an important factor. In fact, the degree of thrombus patency was closely related to HSP expression.

In this study, we performed immunohistochemical search for heat shock proteins, especially HSP27 and HSP70. HSP27 is ubiquitously present in both the cytoplasm and nucleus, and its expression level increases upon exposure to heat shock and various stress conditions. The major roles of HSP27 include regulation of protein folding by chaperone function, protein degradation, maintenance of the cytoskeleton, regulation of the cell cycle, immune response, promotion of cancer, induction of resistance to anticancer drugs, aging, biomarkers of disease, exacerbation of neurodegenerative diseases, development, and differentiation^6–9,27–36^. HSP70s are abundant in cancer cells and have been shown to suppress multiple apoptotic pathways, regulate necrosis, evade cellular senescence programs, interfere with tumor immunity, promote angiogenesis, and even affect tumor metastasis^6,8,30^.

Recently, Thienel et al. reported that distinct antithrombotic patterns were detected in the platelets of hibernating brown bears. Among these, HSP47, which is an essential intracellular chaperone molecule for facilitating collagen production within cells that secrete collagen, showed the most notable decrease. Lowering or eliminating HSP47 led to the inhibition of immune cell activation and the formation of neutrophil extracellular traps, contributing to the prevention of blood clotting in bears, patients with spinal cord injuries, and mice^37^. In addition, HSP47 was reported to be present on the platelet surface, where it interacts with collagen, stabilizes platelet adhesion, and increases collagen-mediated signaling, thus promoting thrombus formation and hemostasis^38^. On the other hand, ex vivo, pharmacological inhibition of Hsp70 in human whole blood prevented the formation of platelet aggregates on collagen under shear^39^. In fact, we found that both HSP70 and HSP27 inhibitors showed reduced thrombi mass in mice. These findings of these HSPs could serve as the foundation for a new protective mechanism against the development of thrombus formation.

We found a relationship between the degree of thrombosis and the expression of HSPs in a DVT model. Although few positive cells and gene expressions were observed until the third day of thrombus formation, both HSP27 and HSP70 increased from the 5-day to the 10-day, suggesting that the stress of IVC ligation and the subsequent initiation of blood coagulation induced these HSPs expressions. The intrathrombotic HSPs were mainly expressed on F4/80^+^ macrophages to promote thrombolysis, and were involved in the formation of intrathrombotic neovessels. Although the expression period of HSPs was short, ranging from 5 to 14 days, HSP27 was expressed in large numbers and clearly, especially on 10-day, and may be a useful indicator to determine the age of thrombosis.

Taken together, our observations implied that both HSP27 and HSP70 might be a molecular target for thrombus therapy from the aspects of clinical relevance. Moreover, the detection of intrathrombotic HSP27 and HSP70 could provide useful information for the estimation of thrombus ages with potential applications in forensics. Collectively, our findings support the interdisciplinary, multifaceted nature of both HSP27 and HSP70.

## Materials and methods

### Stasis-induced deep vein thrombus model

Specific pathogen-free 8- to 10-week-old male BALB/c mice were purchased from SLC (Shizuoka, Japan). Intravenous thrombus was induced as described previously^11–14^. Under the deep anesthesia by isoflurane inhalation (FUJIFILM Wako Pure Chemical Corporation, Osaka, Japan), an incision was made on the abdominal wall, and the inferior vena cava (IVC) was exposed and ligated. At the indicated time intervals, mice were euthanized by the inhalation of over-dose isoflurane, and thrombus samples were obtained. We used 5 mice at each time point. In another series, BALB/c mice were treated intraperitoneally HSP27 inhibitor (50 μg/mouse/day; HY-124653, MedChemExpress, Monmouth Junction, NJ) or HSP70 inhibitor (0.1 mM/mouse/day; A18734, Adooq BioScience, Irvine, CA), or PBS as controls at one day before the IVC ligation and at 1 and 3 days after the IVC ligation. At day 5 after the IVC ligation, intravenous thrombi were harvested for the determination of the weights and blood flow. Five to six mice were used per group. All animal experiments were approved by the Committee on Animal Care and Use of Wakayama Medical University.

### Human deep vein thrombus samples

A total of 7 human thrombus samples were collected from forensic autopsy cases with a postmortem interval (PMI) of less than 72 h. The individual ages ranged from 33 to 90 years (mean age, 65.1 years). Samples were subjected to subsequent analyses.

### Histopathological analysis and immunohistochemistry

The thrombus samples were fixed in 4% formaldehyde buffered with PBS (pH 7.2) for 24 h, and 4-µm-thick paraffin-embedded sections were made^11,40^. For mice thrombi, immunostaining of HSP27 and HSP70 were performed by Ventana Discovery XT (Ventana Medical Systems, Inc., AZ, USA) using mouse anti-HSP27 monoclonal antibodies (mAbs) (sc-13132, Santa Cruz Biotechnology, Inc., Santa Cruz CA, USA), mouse anti-HSP70 mAb (sc-32239, Santa Cruz Biotechnology, Inc., Santa Cruz CA, USA). On the other hand, for human thrombi, the sections were stained with Hematoxylin and eosin (HE), Masson’s Trichrome (MT), and Berlin blue after deparaffinization^12–14,26^. As described previously, immunostaining of HSP27, HSP70, myeloperoxidase (MPO), and CD68 were performed by Ventana Discovery XT using mouse anti-HSP27 mAb (sc-13132, Santa Cruz Biotechnology), mouse anti-HSP70 mAb (sc-32239, Santa Cruz Biotechnology), rabbit anti-MPO pAbs (RB-373-A, Lab Vision/Neo Markers, Fremont, CA), and mouse anti-CD68 mAb (clone PG-M1, M0876, DAKO, Glostrup, Denmark)^11,13,14^. After incubating the specimens with HRP-conjugated anti-mouse multimer (518-102128, OmniMap HRP Multimer, Roche Diagnostics K.K., Tokyo, Japan), immune complexes were visualized using Chromomap kit DAB (518-100803, Roche Diagnostics K.K.) according to the manufacturer’s instructions. The number of cells positive for HSP27^+^ and HSP70^+^ in mice and human thrombi were evaluated semi-quantitatively as described previously^11,14,41^. Immunopositive cells were enumerated in five high-power fields (hpf, ×1000) within the thrombus, the total numbers in the five fields were combined. All measurements were performed by two examiners without prior knowledge.

### Double-color immunofluorescence analysis

Double-color immunofluorescence analysis of HSP27 and F4/80 (MCA497, Bio-Rad Laboratories, Hercules, CA), CD11b (rabbit anti-CD11b mAb, ab133357, clone EPR1344, Abcam, Cambridge, UK), MPO (rabbit anti-MPO pAb, RB-373-A, Lab Vision/Neo Markers), α-SMA (mouse anti-α-SMA mAb, ab7817, clone 1A4, Abcam), or CD31 (rat anti-CD31 mAb, clone MEC 13.3, BD Biosciences, Franklin Lakes, NJ), and HSP70 and F4/80 or CD11b were performed by Ventana Discovery XT^13^. Deparaffinized-sections were incubated in a combination of mouse anti-HSP27 mAb and rat anti-F4/80 mAb or anti-HSP70 mAb and rat anti-F4/80 mAb. After incubation with FITC-labelled (3 μg/ml, Jackson Immunoresearch Laboratories, West Grove, PA) and Cy3-labelled secondary pAbs (0.75 μg/ml, Jackson Immunoresearch Laboratories), the sections were observed by fluorescence microscopy^16,18^. Each section was enclosed using mounting medium for fluorescence with DAPI (H-1200Vectashield, Vector).

### Morphometrical analysis of collagen deposition, histopathological and immunohistochemical staining of human thrombi

We analyzed collagen accumulation, hemosiderin-positive cell counts, and neutrophil/macrophage ratios in seven autopsy thrombi. The most critical event for the formation of thrombi is the rise in collagen content within the thrombus. We have previously reported that measurement of collagen accumulation in mouse thrombus is useful for estimating thrombus age^26^. Therefore, intrathrombotic collagen deposition was semi-quantitated as the blue area in MT-stained sections. The area of collagen deposition in the thrombus was evaluated by Image J analysis software Ver. 1.38w (National Institute of Health, USA), and expressed as the percentage of the whole thrombus area. For semiquantitative evaluation of Berlin blue staining sections, morphometrical analysis was performed, as described previously^26^. Briefly, in each section, five microscopic fields (two central and three peripheral fields) were randomly selected (magnification 1000×), and the number of hemosiderin-positive cells within the thrombi were counted and summed from the five microscopic fields. Moreover, in each section, five microscopic fields were randomly selected (1000×), and the number of MPO-positive neutrophils and CD68-positive macrophages within the thrombi were counted and summed from the five microscopic fields^25^. Thereafter the ratios of neutrophils to macrophages (N/M ratios) were calculated.

### Extraction of total RNAs and real-time RT-PCR

Real-time RT-PCR was performed as described previously^12–14^. Briefly, total RNA was extracted from thrombus tissue samples using ISOGEN (Nippon Gene, Toyama, Japan) according to the manufacturer’s instructions, and 1 μg of total RNAs were reverse-transcribed into cDNA at 42 °C for 1 h in 20 μl reaction mixture containing mouse Moloney leukemia virus reverse transcriptase (PrimeScript, TAKARA BIO, Shiga, Japan) with random 6 primers (TAKARA BIO). Thereafter, generated cDNA was subjected to real-time PCR analysis using TB Green Premix Ex Taq II (TAKARA BIO) with the sets of specific primers (Table 3). Relative quantity of the target gene expression to β-actin gene was measured by comparative Ct method.

**Table 3:** Sequences of the primers used for real-time RT-PCR.

Transcript	Sequences
*Hspb1*	(F) 5′-TCCCTGGACGTCAACCACTTC-3′
	(R) 5′-AGAGATGTAGCCATGTTCGTCCTG-3′
*Hspa1a*	(F) 5′-GTGGTTGCACTGTAGGACTTGTTTC-3′
	(R) 5′-GACCCGAGTTCAGGATGGTTG-3′
*β-actin*	(F) 5′-CATCCGTAAAGACCTCTATGCCAAC-3′
	(R) 5′-ATGGAGCCACCGATCCACA-3′

*F* forward primer, *R* reverse primer.

### Statistical analysis

All data are presented as the mean ± SEM. To compare the values between two groups, Student *t* test was performed. All statistical analyses were performed using Microsoft Excel. *p* < 0.05 was accepted as significant. For human thrombus samples, all data are presented as the mean ± SEM. Statistical significance was evaluated using Mann–Whitney’s *U*-test. *P* < 0.05 was accepted as significant.

### Ethical approval

All animal experiments were approved by the Committee on Animal Care and Use at Wakayama Medical University (No. 1082) and all methods were performed in accordance with relevant regulations and guidelines including the ARRIVE guideline. This study was approved by the Research Ethics Committee of Wakayama Medical University (No. 3314) regarding the use of human samples. All the procedures were performed in accordance with the Declaration of Helsinki Principles. Moreover, this study was conducted using autopsy records from the past, and we could not obtain informed consent from the bereaved family for the use of these records. Therefore, we conducted this study in accordance with the "Ethical Guidelines for Medical Research Involving Human Subjects (enacted by the Ministry of Health, Labor, and Welfare in Japan), section 12–1 (2) (a) (c).” This was a de-identified study using archived tissue obtained from judicial autopsy cases, and the information on the implementation of the study was posted on our website (https://www.wakayama-med.ac.jp/dept/igakubu/160420/index.html). The review board of the Research Ethics Committee of Wakayama Medical University waived the need for written informed consent from the relatives of the individuals studied in accordance with the national legislation and the institutional requirements.

## Data Availability

The authors declare that all data are available in the article file or available from the corresponding author, Toshikazu Kondo, upon reasonable request.
